# Supplementary material for: Classification of patients with COVID-19 by blood RNA endotype: a prospective cohort study
Source: Microbiol Spectr. 2023 Nov 15;11(6):e02645-23. doi: 10.1128/spectrum.02645-23 (PMC10715063; doi:10.1128/spectrum.02645-23)
Supplement: Supplemental table and figures — Table S1; Fig. S1 to S5. [file spectrum.02645-23-s0001.docx]

Supplementary Appendix

**Supplementary tables**

Table S1 Candidate RNAs identified using upstream regulator analysis 2

**Supplementary figures**

Figure S1 Sample size analysis of discovery cohort 4

Figure S2 Volcano plots for the three subcohorts 5

Figure S3 Derivation of candidate RNAs from RNA sequence analysis 6

Figure S4 Derivation of candidate RNAs from RT-qPCR analysis 7

Figure S5 Plasma level of PRL and TLR3 in plasma from O-link analysis 8

| **TABLE S1.** Candidate RNAs identified using upstream regulator analysis | | | | | |
| --- | --- | --- | --- | --- | --- |
| **Upstream regulator** | **Molecular type** | **Predicted**  **activation state** | **Fold change (log2)** | **z-score** | **P value** |
| **COVID-19 patients vs. Healthy volunteers** | | | | | |
| STAT1 | Transcription regulator | Activated | 1.064 | 4.393 | 5.46E-20 |
| IL1B | Cytokine | Activated | 1.063 | 4.815 | 1.46E-18 |
| IRGM | Enzyme | Inhibited | -2.022 | -5.739 | 3.4E-18 |
| OSM | Cytokine | Activated | 2.29 | 4.28 | 4.06E-18 |
| TGM2 | Enzyme | Activated | 0.596 | 7.254 | 1.67E-14 |
| CEBPB | Transcription regulator | Activated | 1.161 | 3.662 | 3.16E-12 |
| SPI1 | Transcription regulator | Activated | 1.659 | 3.055 | 3.73E-12 |
| IRF1 | Transcription regulator | Activated | 0.805 | 5.019 | 3.77E-12 |
| PRL | Cytokine | Activated | 2.999 | 2.371 | 9.39E-12 |
| CEBPA | Transcription regulator | Activated | 0.362 | 3.943 | 1.18E-11 |
| IRF7 | Transcription regulator | Activated | 2.05 | 6.689 | 9.36E-11 |
| FOXM1 | Transcription regulator | Activated | 0.629 | 3.55 | 1.75E-09 |
| CKAP2L | Other | Activated | 2.703 | 4.95 | 3.75E-09 |
| PARP9 | Enzyme | Activated | 1.696 | 2.147 | 4.39E-09 |
| EIF2AK2 | Kinase | Activated | 1.989 | 5.059 | 1.38E-08 |
| IL10RA | Transmembrane receptor | Inhibited | -0.775 | -3.953 | 1.83E-08 |
| STAT2 | Transcription regulator | Activated | 0.5 | 3.738 | 5.35E-08 |
| ERN1 | Kinase | Activated | 0.503 | 2.12 | 6.92E-08 |
| CSF1 | Cytokine | Activated | 0.829 | 2.755 | 2.09E-07 |
| NFKBIA | Transcription regulator | Activated | 1.87 | 2.236 | 8.72E-07 |
| **Critical vs. Non-critical** | | | | | |
| TGFB1 | Growth factor | Activated | 0.489 | 2.291 | 5.03E-13 |
| IL1B | Cytokine | Activated | 0.816 | 3.28 | 8.54E-10 |
| OSM | Cytokine | Activated | 1.176 | 3.49 | 2.28E-09 |
| IL10RA | Transmembrane receptor | Inhibited | -0.448 | -2.072 | 1.3E-08 |
| STAT3 | Transcription regulator | Activated | 0.933 | 3.123 | 1.38E-08 |
| MYD88 | Other | Activated | 0.759 | 2.033 | 1.87E-08 |
| SPI1 | Transcription regulator | Activated | 1.324 | 2.284 | 1.76E-07 |
| NR3C1 | Ligand-dependent nuclear receptor | Activated | 0.347 | 2.258 | 6.56E-07 |
| SELPLG | Other | Activated | 1.048 | 2.538 | 0.00000238 |
| CSF1 | Cytokine | Activated | 0.858 | 2.298 | 0.00000389 |
| USP22 | Peptidase | Activated | 0.208 | 3 | 0.0000191 |
| SELP | Transmembrane receptor | Activated | 0.932 | 2.646 | 0.000149 |
| MAPK14 | Kinase | Activated | 1.419 | 2.282 | 0.000257 |
| CEBPA | Transcription regulator | Activated | 0.663 | 2.643 | 0.000305 |
| TNFSF10 | Cytokine | Activated | 0.327 | 2.075 | 0.00032 |
| EGF | Growth factor | Activated | 0.899 | 3.519 | 0.000618 |
| CEBPE | Transcription regulator | Activated | 0.608 | 2.2 | 0.000872 |
| EIF2AK2 | Kinase | Activated | 0.887 | 2.236 | 0.000912 |
| TNFSF9 | Cytokine | Inhibited | -0.997 | -2.183 | 0.00097 |
| JAK1 | Kinase | Activated | 0.413 | 2.158 | 0.00102 |
| **Early recovery patients: phase 1 vs. phase 2** | | | | | |
| STAT1 | Transcription regulator | Activated | 0.935 | 5.03 | 1.52E-33 |
| IRF7 | Transcription regulator | Activated | 1.814 | 5.709 | 2.23E-33 |
| IRGM | Enzyme | Inhibited | -0.834 | -4.69 | 3.43E-30 |
| IFNG | Cytokine | Activated | 0.605 | 6.047 | 1.04E-24 |
| IFNB1 | Cytokine | Activated | 0.252 | 5.144 | 3.74E-24 |
| IRF1 | Transcription regulator | Activated | 0.444 | 4.911 | 7.39E-23 |
| CNOT7 | Transcription regulator | Inhibited | -0.103 | -2.219 | 3.24E-22 |
| TRIM24 | Transcription regulator | Inhibited | -0.049 | -4.141 | 6.3E-22 |
| STAT2 | Transcription regulator | Activated | 1.08 | 3.067 | 7.29E-22 |
| TGM2 | Enzyme | Activated | 0.915 | 3.709 | 1.14E-19 |
| EIF2AK2 | Kinase | Activated | 1.927 | 4.082 | 1.38E-17 |
| IFNAR1 | Transmembrane receptor | Activated | 0.181 | 2.797 | 1.63E-17 |
| PML | Transcription regulator | Activated | 1.07 | 3.052 | 7.9E-16 |
| STAT6 | Transcription regulator | Inhibited | -0.141 | -3.08 | 1.77E-15 |
| IL1B | Cytokine | Activated | 0.723 | 5.092 | 7.55E-14 |
| ZBTB10 | Other | Activated | 0.242 | 3.77 | 7.68E-14 |
| SPI1 | Transcription regulator | Activated | 0.276 | 3.317 | 1.14E-13 |
| DUSP11 | Phosphatase | Inhibited | -0.22 | -2.97 | 3.39E-13 |
| DNASE2 | Enzyme | Inhibited | -0.446 | -2.416 | 4.74E-13 |
| TLR3 | Transmembrane receptor | Activated | 0.046 | 3.809 | 6.39E-13 |


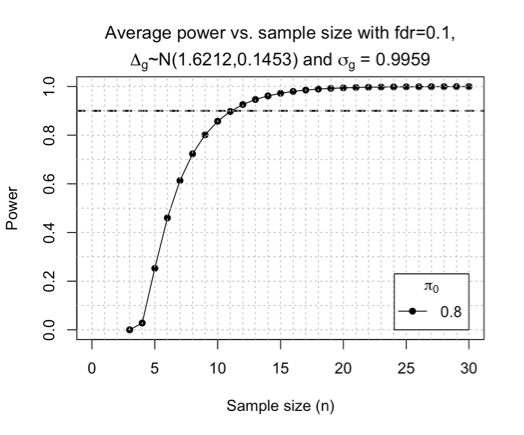


**Figure S1.** Sample size analysis of discovery cohort. The number of samples required to extract candidate RNAs of prognostic relevance was calculated to be at least 12 cases for 90% power with measured RNA = 27,000, |log fold change| = 1.2, and false discovery rate = 0.1.

(a)

(b)


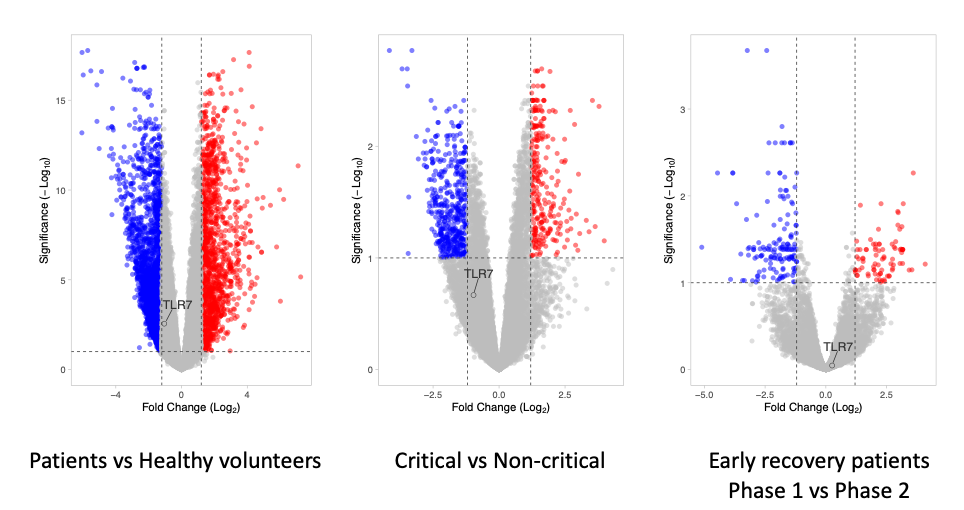


**Figure S2.** Volcano plots for the three subcohorts. Significance was defined as |minimum log2 fold change| >1.2 and false discovery rate <0.1. (a) The RNAs indicated in the panel are the top 20 RNAs based on fold change in expression. (b) The fold change of TLR7 is shown.

**Figure S3.** Derivation of candidate RNAs from RNA sequence analysis. Expression levels of 31 RNAs were significantly changed between critical and non-critical patients. NS, not significant. * P<0.05; ** P<0.01; *** P<0.001.

**Figure S4.** Derivation of candidate RNAs from RT-qPCR analysis. (a) Expression levels of five RNAs (IL18R1, LGALS2, MAPK14, PRL, and TLR3) were significantly changed between critical and non-critical. NS, not significant. (b) Expression levels of five RNAs were significantly elevated in Phase 1 and improved to healthy levels in Phase 2. * P<0.05; ** P<0.01; *** P<0.001.

**Figure S5.** PRL and TLR3 in plasma from O-link analysis. Plasma levels of PRL were significantly increased in both Phase 1 and Phase 2, whereas TLR3 was significantly decreased only in Phase 2. NPX, normalized protein expression. ** P<0.01
