# Supplementary material for: Classification of patients with COVID-19 by blood RNA endotype: a prospective cohort study
Source: Microbiol Spectr. 2023 Nov 15;11(6):e02645-23. doi: 10.1128/spectrum.02645-23 (PMC10715063; doi:10.1128/spectrum.02645-23)
Supplement: Supplemental text — Supplemental methods. [file spectrum.02645-23-s0002.docx]

**Supplemental Methods**

**Cohort Data**

In the discovery cohort, the clinical data of patients were collected from electronic medical records and included sex, age, comorbid conditions, body mass index (BMI), laboratory data at inclusion, PaO_2_/FIO_2_ (P/F) ratio at inclusion, Sequential Organ Failure Assessment (SOFA) score, steroid treatment before inclusion, day from onset to inclusion, and 28-day mortality. The control population in the discovery cohort consisted of healthy volunteers enrolled via public poster advertisements. Blood samples were obtained from the patients on day 1 (day of admission to Osaka University Hospital) and days 6–14 and once from the healthy volunteers. RNA expression in whole-blood samples was evaluated using RNA sequencing (RNA-seq).

Based on the results of the statistical analysis of RNA-seq data of COVID-19 patients and healthy volunteers, RT-qPCR using Biomark™ HD (Fluidigm) was performed for 31 RNAs (ALOX15, CD177, CEBPB, CYP19A1, ERN1, FCER1A, HDC, IFNAR1, IFNG, IL18R1, IL1R2, IRF1, LGALS2, LINC00676, LINC01093, LRRC70, LRRN1, LRRN3, MAPK14, MYD88, NFKBIA, OLAH, OSM, PARP9, PID1, PRL, SELPLG, SPI1, STAT1, STAT3, and TLR3).

In the validation cohort, demographic variables of all patients including sex, age, comorbid conditions, BMI, laboratory data at inclusion, P/F ratio at inclusion, SOFA score, steroid treatment before inclusion, day from onset to inclusion, and 28-day mortality were collected. Blood samples were collected from the patients until discharge from the hospital or death on day 1 (day of admission at the Osaka University Hospital or the Osaka General Medical Center) and on days 6–14. The blood samples were stored at -30°C until further analysis. Whole-blood RNA expression was investigated using the technique (Biomark HD) as used for the discovery cohort. Laboratory tests of the blood samples from the patients admitted with COVID-19 were systematically performed by the central laboratory at each hospital.

In the protein profile cohort, the clinical data were collected from electronic medical records and included sex, age, comorbid conditions, BMI, laboratory data at inclusion, P/F ratio at inclusion, SOFA score, steroid treatment before inclusion, day from onset to inclusion, and 28-day mortality. The control population consisted of healthy volunteers enrolled via public poster advertisements. Blood samples were obtained from the patients on days 1 (day of admission at Osaka University Hospital) or 2 and days 6–8 and once from the healthy volunteers. Plasma proteomics were performed by using O-link^®^ Explore 1536.

**RNA-seq**

Total RNA was isolated from the leukocytes using a PAXgene Blood RNA System (BD Bioscience). Full-length cDNA was generated using a SMART-Seq HT Kit (Takara Bio, Mountain View, CA) according to the manufacturer’s instructions. An Illumina library was prepared using a Nextera DNA Library Preparation Kit (Illumina) according to SMARTer kit instructions. DNA libraries were converted to libraries compatible for DNBSEQ using an MGIEasy Universal Library Conversion Kit (App-A). Sequencing was performed on a DNBSEQ-G400RS platform in 2×100-bp paired-end mode.

The sequenced reads were mapped to the human reference genome sequences (hg19) using TopHat, version 2.0.13, in combination with Bowtie2, version 2.2.3, and SAMtools, version 0.1.19. The fragments per kilobase of exons per million mapped were calculated using Cufflinks, version 2.2.1. Raw read counts of gene-level expression were calculated using featureCounts, and relative RNA expression levels were calculated using the DESeq algorithm.

**Reverse-transcription quantitative PCR (RT-qPCR)**

Pre-amplification of the target genes from the healthy volunteers and cDNA samples from the COVID-19 patients was performed as per the Fluidigm protocol. Briefly, a single primer pool (500 nM concentration of each primer) was created by mixing 1 μL of each target gene primer (forward and reverse) with 152 μL of nuclease-free water (Integrated DNA Technology; 100 μM stocks). Next, a master mix was prepared, containing 105.6 μL of PreAmp Master Mix (Fluidigm), 132.0 μL of the 500-nM primer pool, and 158.4 μL of nuclease-free water. We added 3.75 μL of the master mix and 1.25 μL of the appropriate cDNA samples to a 96-well plate. The PCR products were amplified using a SimpliAmp Thermal Cycler (Thermo Fisher Scientific) under the following conditions: initial denaturation at 95°C for 2 min, followed by 14 cycles of denaturation at 95°C for 15 s and annealing at 60°C for 4 min.

The PCR products were treated with exonuclease I to remove all components of the pre-amplification reaction. A master mix containing 24 μL of Exonuclease I 10X Reaction Buffer (New England Biolabs), 48 μL of Exonuclease I (New England Biolabs), and 168 μL of nuclease-free water was prepared, and 2 μL of the master mix was added to each well of the 96-well PCR plate containing preamplification samples. The exonuclease digestion was performed in a SimpliAmp Thermal Cycler as per Fluidigm protocols. After the digest reaction, the PCR plate was placed at -80°C until further analysis.

The 48.48 IFC chip was primed as per the manufacturer’s instructions using 300 μL of control line fluid (mineral oil) in a JunoTM System (Fluidigm). We prepared primer mixes (concentration of each mix: 5 μM) for seven target genes by mixing 25 μL of 2X assay loading reagent (Fluidigm), 22.5 μL of DNA suspension buffer (Teknova), and 2.5 μL of a pre-prepared 100 μM primer stock. A primer mix without any primers was used as the no-primer control. A master mix was prepared by mixing 180 μL of 2X SSO Fast™ Eva Green qPCR master mix (Bio-Rad) with reference dye (Integrated DNA Technology) and 18 μL of 20X DNA binding dye (Fluidigm).

Next, we added 5 μL of the prepared primer mixes and 5 μL of the sample mixes to their relevant inlets in the 48.48 IFC chip; 2.75 μL of the master mix and 2.25 μL of exonuclease-treated pre-amplified cDNA samples or nuclease-free water were added to each well. This was followed by priming the chip using the Juno System (Fluidigm) as per the manufacturer’s instructions. Finally, the chip was loaded onto the instrument for qPCR. The PCR conditions were as follows: hot start at 95°C for 1 min, followed by 20 cycles of denaturation at 95°C for 5 s, annealing at 58°C for 20 s, and then melt curve analysis from 58 to 95°C with 3 s between each increment of temperature.

All retrieved qPCR data were analyzed using Fluidigm Real-Time PCR Analysis software ver 4.5.2. The housekeeping gene GAPDH was assigned as the reference gene, and delta-delta Ct values (ΔΔCt) and fold change values of the selected genes from both cohorts were calculated based on the values for the housekeeping gene.

**Olink multiplex proximity extension assay and Olink^®^ Explore 1536**

The Olink multiplex proximity extension assay is a dual-recognition immunoassay in which two matched antibodies labelled with unique DNA oligonucleotides are used to bind target proteins. Upon binding, the oligonucleotides come in close proximity and hybridize followed by extension that generates a unique sequence used for digital identification of the specific protein assay (www.olink. com). A volume of 2.8 µL of plasma with oligonucleotide-labeled antibody pairs to form specific DNA duplexes was incubated overnight. Then, after combined extension and pre-amplification, the individual protein markers were measured using a Nova-Seq 6000 system. The resulting counts were normalized against an extension control and an inter-plate control. The levels of the proteins are expressed as normalized protein expression (NPX) values in log2 scale.
